# Concurrent Use in Taiwan of Chinese Herbal Medicine Therapies among Hormone Users Aged 55 Years to 79 Years and Its Association with Breast Cancer Risk: A Population-Based Study

**DOI:** 10.1155/2014/683570

**Published:** 2014-05-29

**Authors:** Yueh-Ting Tsai, Jung-Nien Lai, Chien-Tung Wu, Shun-Ku Lin

**Affiliations:** ^1^Institute of Traditional Medicine, School of Medicine, National Yang-Ming University, No. 155, Section 2, Linong Street, Beitou District, Taipei City 112, Taiwan; ^2^Department of Chinese Medicine, Taipei City Hospital, Yangming Branch, No. 105, Yusheng Street, Shilin District, Taipei City 111, Taiwan; ^3^Taiwan Association for Traditional Chinese Medicine of Family, 9F., No. 105, Yusheng Street, Shilin District, Taipei City 111, Taiwan; ^4^Department of Chinese Medicine, Taipei City Hospital, Renai Branch, No. 10, Section 4, Ren'ai Road, Da'an District, Taipei City 106, Taiwan

## Abstract

*Background*. The purpose of the present study was to analyze the concurrent use of Chinese herbal products (CHPs) among women aged 55 to 79 years who had also been prescribed hormonal therapies (HT) and its association with breast cancer risk. *Methods*. The use, frequency of service, and CHP prescribed among 17,583 HT users were evaluated from a random sample of 1 million beneficiaries from the National Health Insurance Research Database. A logistic regression method was used to identify the factors that were associated with the coprescription of a CHP and HT. Cox proportional hazards regressions were performed to calculate the hazard ratios (HRs) of breast cancer between the TCM nonusers and women who had undergone coadministration of HT and a CHP or CHPs. *Results*. More than one out of every five study subjects used a CHP concurrently with HT (CHTCHP patients). *Shu-Jing-Huo-Xie-Tang* was the most commonly used CHP coadministered with HT. In comparison to HT-alone users, the HRs for invasive breast cancer among CHTCHP patients were not significantly increased either in E-alone group or in mixed regimen group. *Conclusions*. The coadministration of hormone regimen and CHPs did not increase the risk of breast cancer.

## 1. Introduction

The results of the observational Million Women Study (MWS) [1], the Nurses' Health Study (NHS) [2], and a random sample of a nation-wide representative cohort of 65,723 Asian women [3] suggest that there is an association between estrogen plus progesterone and breast cancer during routine gynaecology practice [4]. Since the WHI published their major findings suggesting that estrogen plus progestin may stimulate breast cancer growth and hinder breast cancer diagnosis in 2003, due to fear of any challenge from patients and/or their families, physicians typically do not prescribe hormones to any patient if there is any doubt regarding the possible beneficial effect or there is a potential harmful effect of the hormones [5–7]. Specifically, the National Health Insurance in Taiwan stipulates a three-month upper limit for repeat prescriptions, which provides a constraint on any unnecessary or harmful medication use; nonetheless, a positive association between estrogen plus progesterone and the occurrence of invasive breast cancer in women in Taiwan can still be observed under the above circumstances [3]. Not surprisingly, many climacteric women have turned to traditional Chinese medicine (TCM) remedies to manage their symptoms; this is because they believe that such treatments have less subjective residual effects.

Although TCM remedies are promoted as natural and therefore harmless, such complementary and alternative medicines are also used in Western countries [8, 9]. In this context, information is limited regarding their safety when used in combination with hormonal therapies (HT). This is especially true regarding the risk of herb-drug interactions, such as interference with the clearance of either of the drugs. Studies on the prevalence of HT use and the coprescription patterns of TCM remedies among climacteric women are rare.

Comprising unique traditional therapies for various ailments, TCMs have been used in Taiwan for hundreds of years, and their popularity remains unabated, despite the present availability of modern medical care in Taiwan. In addition, one distinguishing feature of the national health care system in Taiwan is the coexistence of modern Western medicine (WM) and TCM, which includes acupuncture and manipulative therapies as well as Chinese herbal products (CHPs); claims for all of the above three aspects of TCM have been covered by the National Health Insurance (NHI) system since 1995 [10].

People in Taiwan are free to choose from care offered by WM clinics or by TCM clinics. With an insured rate of 98% to 99%, the random sample that comprises the NHIRD is representative of the general population of Taiwan and should allow a reasonably accurate assessment of the coutilization of TCM and modern medical resources in Taiwan. Therefore, the NHI research database (NHIRD) provides an ideal platform for pharmacoepidemiological studies [11]. In this context, our study aimed, using a nationwide cohort from Taiwan, to describe the demographics and patterns of CHP usage with respect to HT users and to explore the risk of breast cancer among HT users. Our findings provide evidence-based information that will help the formulation of appropriate management strategies in relation to drug safety and integrative medicine.

## 2. Material and Methods

### 2.1. Data Source and Participants

Our study protocols were approved by the Institutional Review Board of the Taipei City Hospital. Our population-based study retrospectively analyzed the reimbursement records of one million NHI beneficiaries from the NHIRD that had been previously selected at random from the 22 million beneficiaries of the NHI; the aim was to determine the prevalence in Taiwan of concurrent CHP and HT and its association with breast cancer risk between January 1, 1997, and December 31, 2008. The electronic records of the NHIRD used encrypted identification numbers for all beneficiaries and are transformed and maintained by the National Health Research Institutes (NHRI) of Taiwan [10, 12].

The NHIRD records contained demographic information, including age and sex, together with clinical data, including all records of clinical visits and hospitalizations, as well as all information regarding prescribed drugs and dosages, which included CHPs. The diagnoses used in the NHIRD were coded according to the International Classification of Diseases, Ninth Revision, Clinical Modification (ICD-9-CM) [13].

To obtain a consistent cohort, all women aged 55 to 79 years who used hormonal drugs, namely, estrogen and progesterone, were reviewed. For the purpose of studying the use of CHPs, we downloaded the claims forms for reimbursed CHPs from the website of the Department of Chinese Medicine and Pharmacy, Ministry of Health and Welfare, Taiwan (DCMP), including the name of each herb, the proportion of each constituent of the mixture, the date and period of approval of the drug, the manufacturer code, and the name of the CHP manufacturer. All CHPs with the same DCMP standard formula were classified in the same categories, regardless of slight variations among the products from different CHP manufacturers [12]. For our analysis of the demographic and clinical variables, a coprescription of a CHP and a HT (defined as prescriptions for both that were issued either simultaneously or issued separately but with overlapping treatment periods) was used to determine the use of a CHP and a hormonal drug on the same day.

**Figure 1:**
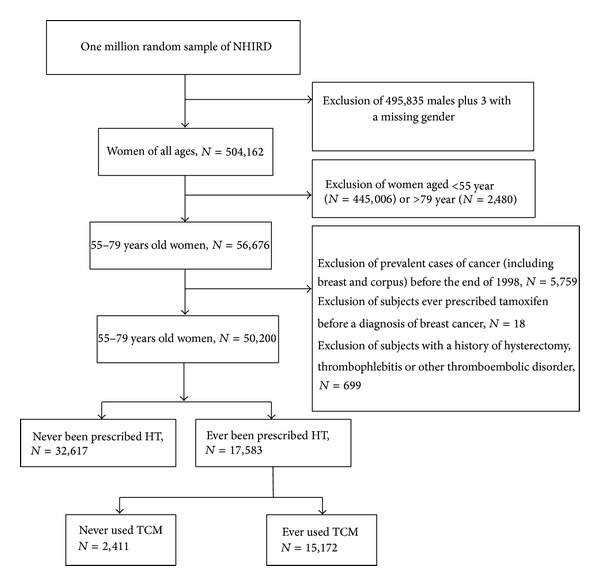
Flowchart of the recruitment of subjects who underwent hormone therapy from the 1 million random sample of the National Health Insurance Research Database (NHIRD) from 1997 to 2008 in Taiwan.

The selection of study subjects from the random sample of one million individuals was performed as follows (Figure 1). First, we excluded all of the male subjects (*n* = 495,835) or any with missing information concerning gender (*n* = 3). Age was calculated by subtracting the subject's birthday from the 1st day of July for each year. Second, subjects under 55 (*n* = 445,006) or over 79 years of age (*n* = 2,480) were excluded to limit the study sample to climacteric women in Taiwan. We then excluded two years, 1997 and 1998, to avoid the inclusion of 5,759 prevalent breast and corpus cancer cases. To control for potential confounding factors, we further excluded 18 subjects who had ever used tamoxifen prior to any diagnosis of gynecological cancer and 699 subjects who had a history of hysterectomy, thrombophlebitis, or thromboembolic disorder.

### 2.2. Study Variables

To identify the key factors associated with the coprescription of CHP and HT among climacteric women, we used demographic factors that had been explored in previous studies [3, 11, 14]. Patients were classified based on age into one of seven groups as follows: 55–59 years, 60–64 years, 65–69 years, 70–74 years, 75–79 years, 70–79 years, and ≥80 years. The geographic areas of Taiwan in which patients resided were used to classify them into one of the seven regions, namely, Taipei City, Kaohsiung City, Northern Taiwan, Central Taiwan, Eastern Taiwan, Southern Taiwan, and the offshore island region. Patients' monthly income in New Taiwan Dollars (NT$) was categorized into one of the following four levels: $0, $1–$19,999, $20,000–$39,999, and ≥$40,000.

### 2.3. HT Exposure Assessment

A total of 17,583 women aged 55 to 79 years were prescribed at least one type of HT during the study period from January 1, 1997, to December 31, 2008. All of the prescribed medications were covered under the NHI of Taiwan and no drugs were dispensed at a pharmacy without a physician's prescription. The reimbursement database contained all the details regarding the prescribed conventional medicines, which included all the various types of HT together with the commercial names of fourteen types of estrogen-containing drugs and ten types of progesterone-containing drugs. The variables for HT usage that were included in the analyses were defined according to the specific proprietary preparation of HT used by the subjects during the study period. We categorised the types of preparations used as follows: estrogen-alone (E-alone); estrogen together with progesterone (E + P); other preparations, which included progesterone only together with vaginal and other local treatments as well as combinations of the above preparation types. To estimate the impact of concurrent use of HT and a CHP on the rate of breast cancer, we selected subjects who were first recorded with a diagnosis of breast cancer (ICD-9-CM codes 1740, 1741, 1742, 1743, 1744, 1745, 1746, 1748, and 1749) between January 1, 2002, and December 31, 2008. Furthermore, we also analyzed the risk of breast cancer according to the period of time patients had been administered HT (current use, last use 1–3 years ago, last use 4-5 years ago, last use ≥ 6 years ago).

### 2.4. Statistical Analysis

Data analysis was conducted using descriptive statistics, including the prescription rates of the patients' concurrent use of a CHP and a HT as stratified by age, the indications for the prescribed CHP and the most frequently coprescribed herbal formulae among the HT users. The incidence rate was summarised as the number of new invasive breast cancer patients/10^3^ person-years at risk. The indications were coded according to the ICD-9-CM and grouped into different broad disease categories. The ICD-9-CM codes 460–519 were classified as diseases of the respiratory system. Codes 780–799 were grouped as symptoms, signs, and ill-defined conditions, and codes 520–579 were classified as diseases of the digestive system. We constructed univariate Cox proportional hazard models to estimate the hazard ratios and their 95% confidence intervals (CI) for new occurrences of invasive breast cancer as shown in Table 3. To minimise the potential confounding by indications for HT, we conducted multivariate Cox regression using current HT users as the reference group to calculate the hazard ratio (HR) for breast cancer among HT users who used a CHP concurrently with HT and HT users who did not use any Chinese herbs after taking age and number of chronic disease into consideration (Table 4). An estimate with a 95% CI that did not contain the number 1 was considered statistically significant. The SAS statistical software, version 9.3 (SAS Institute, Cary, NC, USA), was used for the data management and analysis.

## 3. Results

### 3.1. Demographic Characteristics of Hormone Users Aged 55–79 Years

The database of outpatient claims contained information on 50,200 women aged 55–79 years over the 1997–2008 period. A total of 17,583 climacteric women from this group were treated with a hormonal therapy and were included in our study. Most of these patients used a mixed type of hormonal therapy (HT). Most of the HT users (86.3%) also sought care from TCM practitioners. Among them, up to 20.1% (*n* = 3,053) of the HT users received a coprescription for a CHP, resulting in the coadministration of a HT with a CHP. Such patients who were coadministered the two types of drug are hereafter referred to as CHTCHP patients. The HT users who did not use a CHP were significantly older than the CHTCHP patients. More CHTCHP patients had income levels of $1 to $19,999 and above, resided in Central Taiwan, used a mixed type of HT, had longer mean duration of follow-up, and had lower breast cancer incidence than HT users who did not use a CHP. The adjusted ORs (aORs) and 95% CIs, as calculated using the logistic regression model, are presented in Table 1. Compared with patients aged 50–59 years (aORs = 1.00), the aORs of the CHTCHP patients decreased with age. Patients who used mixed type HT (OR = 2.17; 95% CI: 1.93–2.44) and with more chronic diseases (one chronic disease: OR = 1.58; 95% CI: 1.17–2.14, two: OR = 2.04; 95% CI: 1.53–2.74, and ≥ three: OR = 2.63; 95% CI: 2.01–3.45) were more likely to be CHTCHP patients than were the patients who used E-alone HT or who had no chronic disease.

### 3.2. The Top Five Coprescribed Chinese Formulae among Hormone Users

The details of the most frequently coprescribed CHP or HT are shown in Table 2. The mixed type of HT and* Shu-jing-huo-xie-tang *were the most common HT and CHP, respectively, among the coprescribed pairs.

### 3.3. Hazard Ratios for Breast Cancer among Hormone Users

Tables 3 and 4 summarise the different levels of invasive breast cancer risk for the different types of HT (E-alone, E + P, mixed type, and progesterone-alone) between the CHTCHP patients and the HT users who did not use Chinese herbs. Among the E-alone group, the HR for invasive breast cancer among current CHTCHP patient was 0.30-fold (95% CI: 0.05–1.81) that of the comparison subjects. The HR for the development of invasive breast cancer was slightly increased by 1.21-fold (95% CI: 0.08–19.41) for CHTCHP patient who no longer received E-alone HT for at least four years, by 0.39-fold (95% CI: 0.13–1.24) for current users of mixed regimen, and by HR = 2.87 (95% CI: 0.36–22.98) for patients who had not received the mixed regimen for at least one year. When the reference group comprised current E-alone users, the adjusted HRs for the development of invasive breast cancer were significantly decreased for CHTCHP patient who no longer received E-alone HT for at least one year. The adjusted HR for the development of invasive breast cancer was slightly decreased by 0.91-fold (95% CI: 0.38–2.15) when the concurrent use of HT and CHP had not been used for 1 to 3 years. In general, in comparison to HT-alone users, the HRs for invasive breast cancer among CHTCHP patients were not significantly increased either in E-alone group or mixed regimen group.

## 4. Discussion

With rapidly increasing health care expenditure in Taiwan and other developed countries, which has gone on for some time and is continuing, concerns are growing regarding the benefits and risks of the concurrent use of hormonal prescriptions and herbal remedies. However, verification and quantification of the research and public health implications of these concerns have been limited due to an absence of comprehensive information on exposure to the full range of hormonal therapies and herbal remedies in HT users. According to our review of the literature, this study is the first to use a random population-based cohort to document the coprescription of HT and CHPs in climacteric women with the aim of providing such information.

The present findings confirm that HT use is common among climacteric women and that large numbers of HT users in Taiwan take large quantities of CHPs, as shown in Figure 1. From 1997 to 2008, more than 86% of HT users took at least one CHP at least and 20% of them were identified as having undergone coadministration of a hormonal drug and a CHP on the same day. These rates translate into 348,956 and 70,219 climacteric women, respectively, in Taiwan. In this study we included patients from a random sample of the population-based NHI database who were prescribed HT by qualified conventional physicians; this database has consistently maintained a rate of insured individuals above 96% since 1997. As a result the possibility of recall or selection bias can be excluded. We observed that women who were prescribed the multiple types of HT were more likely to concurrently use a CHP compared with patients who did not seek TCM treatment. This suggests that HT may be failing to fulfill some women's expectations in terms of a complete menopausal care plan [15, 16] and our findings seem to support this to some extent. Furthermore, the increasing incidence of the hormonal-CHP coadministration that we observed may highlight an ignorance of the true nature of CHPs that is held by many HT users. It is of concern that many climacteric women believe it is safe to combine CHPs and prescribed hormonal drugs, notwithstanding that this may result in a possible herb-drug interactions and lead to unpredictable efficacies of the medicines. Therefore, we suggest that a more critical attitude toward the use of HT and CHPs in combination is needed among both physicians and HT users.

The present findings show that nearly nine out of ten HT users aged 55 to 79 years suffering from at least one chronic illness condition and those aged 55–59 years and with multiple chronic illness conditions are more likely to consume CHPs and hormonal therapies concurrently than those in other age groups and those without chronic disease, as shown in Table 1. Symptoms, signs, and ill-defined conditions were the most common reasons for consuming CHPs and hormonal therapies concurrently, followed by “diseases of the respiratory system.” Further analysis found that HT users tended to coadminister HT and Chinese herbal remedies that target menopausal-related symptoms. The climacteric experience involves a complex interaction between sociocultural, psychological, and environmental factors, as well as the biological changes that are related to altered ovarian hormone status and hormone deficiency [17–20]. The above findings corroborate the results of some earlier studies that, in an independent or synergistic way, have shown that menopausal-related symptoms, together with chronic diseases, are able to act with physical, social, and environmental factors to predispose individuals to psychological symptoms [17, 21]. This then results in a certain amount of self-medication with herbal therapies in an attempt to mitigate some of menopausal-related symptoms or the adverse effects of hormonal therapies, without the individual knowing that the combination might actually result in an increased risk [22].

*Shu-jing-huo-xie-tang* was the most commonly coprescribed herbal formula for HT users, as shown in Table 2. According to an ancient TCM book,* Shu-jing-huo-xie-tang* is able to dispel blood stasis and wind-dampness in the “Channels” of the lower part of the body and the abdomen. Despite its high frequency of prescription by TCM practitioners in Taiwan, there has not yet been any clinical trial to demonstrate its efficacy and safety. Our previous clinical trials have demonstrated that* Du-huo-ji-sheng-tang* and* Jia-wei-xiao-yao-san *(Augmented Rambling Powder), the two CHPs that are most commonly coprescribed with HT, after* Shu-jing-huo-xie-tang*, may be an efficacious therapy with respect to reducing pain and stiffness of the knee joint [23, 24] and improving sleep quality [25], respectively. Other formulae commonly coprescribed with HT are associated with relieving low back pain (*Liu-wei-di-huang-wan*) and suppressing palpitations (*Zhi-gan-cao-tang*). It is apparent from this study that TCM doctors in Taiwan prescribe herbal therapies mainly to reduce musculoskeletal discomfort, insomnia, and palpitation, which are the same menopausal symptoms for which HT is prescribed by conventional doctors. Although clinical studies on various herbs have shown promising effects and herbal medicine has been prescribed safely by professionals in the USA and Taiwan for many years, there is yet insufficient evidence to allow a conclusion to be reached regarding the rational use of the aforementioned formulae among HT users.

Since the WHI published their major findings suggesting that estrogen plus progestin may stimulate breast cancer growth and hinder breast cancer diagnosis in 2003, Taiwanese women have been found to be undergoing a period of reduced prescription hormone intake [5]. People in Taiwan are free to choose from the care offered either by WM clinics or by TCM clinics and this NHI policy might result in a certain proportion of the coadministration of a hormonal drug and a CHP on the same day. The present results support the idea that TCM physicians in Taiwan encourage HT users to coadminister CHPs and hormonal therapies in order to relieve their menopausal-related symptoms or to reduce the risk of hormonal adverse effects. In this study, we found that the coadministration of hormone regimen and CHPs had lower incidence rate and did not increase the risk of breast cancer, compared with the users of the hormone regimen who never use Chinese herbs. Importantly, before drawing any conclusions from the findings, further studies, including a joint analysis of breast cancer and the coprescription patterns of particular Chinese herbs and hormonal drugs, are warranted. Therefore, health care providers and public-health policy analysts need to pay greater attention to this particular health care-seeking behavior and should assess the potential long-term impact of coprescribing HT and CHPs on the health outcomes among climacteric women.

Our study has some limitations. First, the NHI only reimburses patients for the cost of the CHP. The cost of the decoction is not reimbursed, which may have affected patients' decisions to use CHPs. Thus, the frequency of concurrent use of HRT and CHPs may be underestimated in our results. However, because the NHI provides comprehensive coverage and the copayment for prescriptions is always $50 (approximately equal to US$1.50), which is generally less than the cost of herbs sold in Taiwan's markets, the likelihood that patients purchased herbs outside of the NHI system is low. Second, we were unable to draw any conclusions regarding the relationship between the severity of the climacteric symptoms and TCM usage because such clinical data are not included in the NHIRD. Third, because the reimbursement data did not include the selection patterns of phytoestrogen-rich foods and the relative weight and reproductive history of the women, we were unable to control for this factor in the model construction. Because the present study included hormone users from a random sampling cohort, we assumed that such confounding factors would not bias the results. Fourth, the retrospective design of our study and the lack of data on dietary and other lifestyle factors may have diminished the statistical power of our findings. Thus, more epidemiological data on occupational exposure, environmental exposure, lifestyle, and medical history are needed to clarify causality.

## 5. Conclusion

This study indicates that further work is required to investigate the implications of CHP use, especially the extent to which the HT users used CHPs concurrently. Our results suggest that, with an equal availability of conventional medical and TCM care, more than one-fifth of the HT users used CHPs concurrently for the relief of climacteric symptoms. Recognizing the benefits of TCM, exploring the potential interactions and adverse effects of TCM, and integrating both health care technologies may be beneficial to the overall health and quality of life of climacteric women, especially those with a family history of breast cancer. Pending further research, we are able to make several recommendations. Firstly, when counseling climacteric women, TCM practitioners must carefully consider the risks and benefits of each Chinese herb and whether hormonal therapy is already being taken by each individual. Secondly, education initiatives are needed to promote a greater awareness of this particular healthcare-seeking behavior and to advise doctors on the possible pitfalls of CHP use. Finally, health care providers should proactively explore a personalized and optimal healthcare for menopausal women, while still attending to their psychosocial and physical needs.

## Figures and Tables

**Table 1:** Demographic characteristics and results of multiple logistic regression showing the adjusted odds ratio (aOR) and 95% CI (confidence interval) of HT* users from the 1  million random sample of the National Health Insurance Research Database (NHIRD) from 1997 to 2008 in Taiwan.

Characteristics	HT users who did not use Chinese medicine, number	HT users coprescribed a Chinese medicine, number	HT users coprescribed a Chinese medicine/HT users who did not use a Chinese medicine aOR (95% CI)
Numbers of HT users (breast cancer patients)	2,411 (31)	3,053 (33)	
Incidence of breast cancer^1^	1.6	1.1	
Mean (SD) duration of follow-up, person-years	8.2 (2.7)	9.4 (2.0)	
Mean (SD) age at inclusion, years	63.4 (6.5)	61.0 (5.2)	
Age groups at inclusion, years number (%)			
55~59	865 (35.9)	1,474 (48.3)	1.00
60~64	607 (25.2)	886 (29.0)	0.83 (0.72–0.96)
65~69	438 (18.2)	446 (14.6)	0.57 (0.48–0.67)
70~74	331 (13.7)	193 (6.3)	0.34 (0.27–0.42)
75~79	170 (7.0)	54 (1.8)	0.21 (0.15–0.29)
$NT^e^/month (Premiums) (%)			
0	768 (31.9)	878 (28.8)	1.00
1–19,999	1,357 (56.3)	1,693 (55.4)	0.95 (0.83–1.09)
20,000–39,999	207 (8.6)	363 (11.9)	1.12 (0.90–1.39)
>=40000	79 (3.3)	119 (3.9)	0.93 (0.67–1.28)
Insured area (%)			
Taipei City	533 (22.1)	562 (18.4)	1.00
Kaohsiung City	162 (6.7)	204 (6.7)	1.16 (0.90–1.49)
Northern Taiwan	647 (26.9)	725 (23.8)	1.06 (0.90–1.26)
Central Taiwan	258 (10.7)	722 (23.6)	2.83 (2.32–3.45)
Southern Taiwan	701 (29.1)	718 (23.5)	1.04 (0.88–1.24)
Eastern Taiwan	95 (3.9)	103 (3.4)	1.04 (0.75–1.44)
Outlying islands	15 (0.6)	19 (0.6)	1.75 (0.80–3.82)
Number of chronic diseases (%)			
0	172 (7.1)	103 (3.4)	1.00
1	360 (14.9)	344 (11.3)	1.58 (1.17–2.14)
Cardiovascular disease	46 (1.9)	38 (1.2)	
Hypertension	89 (3.7)	47 (1.5)	
Hypercholesterolemia	21 (0.9)	20 (0.7)	
Diabetes mellitus	33 (1.4)	24 (0.8)	
Stroke	10 (0.4)	6 (0.2)	
Osteoporosis	161 (6.7)	209 (6.9)	
2	458 (19.0)	528 (17.3)	2.04 (1.53–2.74)
3 or more	1,421 (58.9)	2,078 (68.1)	2.63 (2.01–3.45)
Types and prescription patterns of HT (%)			
Estrogen-alone	1,179 (48.9)	925 (30.3)	1.00
Progesterone-alone	83 (3.4)	0 (0.0)	—
Estrogen and progesterone combination	34 (1.4)	33 (1.1)	1.17 (0.71–1.95)
Mixed type^†^	1,115 (46.3)	2,095 (68.6)	2.17 (1.93–2.44)

*HT refers to hormonal therapy.

^†^Mixed type refers to the estrogen-alone (E-alone); estrogen together with progesterone (E + P); other preparations, which included progesterone only and vaginal and other local treatments and combinations of the above preparation types.

^e^NT$ refers to new Taiwan dollars, of which 1 US $ = 30 NT$.

^1^Incidence rate: number of breast cancer/1,000 person-year.

**Table 2:** The top five coprescribed Chinese formulae among HT users between 1997 and 2008.

Chinese medicine-HRT	Total days of coprescribing	Total people of coprescribing	Average days of coprescribing Chinese and Western medicine (days/people)
Total			
Formulae	131,597	2,569	51.2
*Shu-Jing-Huo-Xue-Tang* (Channel-Coursing Blood-Quickening Decoction)	3,700	322	11.5
*Du-Huo-Ji-Sheng-Tang* (Pubescent Angelica and Mistletoe Decoction)	3,508	271	12.9
*Jia-Wei-Xiao-Yao-San* (Supplemented Free Wanderer Powder)	3,036	249	12.2
*Liu-Wei-Di-Huang-Wan* (Six-Ingredient Rehmannia Pill)	2,960	237	12.5
*Zhi-Gan-Cao-Tang* (Honey-Fried Licorice Decoction)	2,821	179	15.8

**Table 3:** Number (no.) of new cases, population-at-risk, hazard ratios (HR), and 95% confidence intervals (CI) for breast cancer estimated using the univariate Cox regression model on a random sample from the National Health Insurance Research Database among sample subjects and followed from 1997 to 2008.

Presence of breast cancer during the follow-up period	HT users who did use a Chinese medicine. Number of cases/population	HT users coprescribed a Chinese medicine, Number of cases/population	HT users coprescribed a Chinese medicine/HT users who did not use a Chinese medicine HR (95% CI)
HT* use at baseline			
Estrogen-alone			
Current users	3/53	2/117	0.30 (0.05–1.81)
Last use 1–3 years previously	0/211	3/249	—
Last use 4-5 years previously	1/192	1/157	1.21 (0.08–19.41)
Last use >=6 years previously	2/710	1/395	0.90 (0.08–9.91)
Progesterone-alone			
Current users	3/9	0/0	—
Last use 1–3 years previously	0/23	0/0	—
Last use 4-5 years previously	0/19	0/0	—
Last use >=6 years previously	0/32	0/0	—
Estrogen and progesterone combination			
Current users	0/0	0/0	—
Last use 1–3 years previously	0/0	0/0	—
Last use 4-5 years previously	0/0	0/0	—
Last use >=6 years previously	0/0	0/0	—
Mixed type^†^			
Current users	5/69	7/246	0.39 (0.13–1.24)
Last use 1–3 years previously	1/69	8/192	2.87 (0.36–22.98)
Last use 4-5 years previously	0/118	0/614	—
Last use >=6 years previously	16/836	11/1,081	0.53 (0.25–1.15)

*HT refers to hormonal therapy.

^†^Mixed type refers to the estrogen-alone (E-alone); estrogen together with progesterone (E + P); other preparations, which included progesterone only and vaginal and other local treatments and combinations of the above preparation types.

**Table 4:** Number (no.) of new cases, population-at-risk, hazard ratios (HR), and 95% confidence intervals (CI) for breast cancer estimated using the multivariate Cox regression model on a random sample from the National Health Insurance Research Database among sample subjects and followed from 1997 to 2008.

Presence of breast cancer during the follow-up period	HT users who did use a Chinese medicine. Number of cases/population	HT users coprescribed a Chinese medicine, Number of cases/population	HT users coprescribed a Chinese medicine/HT users who did not use a Chinese medicine aHR (95% CI)
HT* use at baseline			
Estrogen-alone			
Current users	3/53	2/117	1.00
Last use 1–3 years previously	0/211	3/249	0.22 (0.05–0.93)
Last use 4-5 years previously	1/192	1/159	0.19 (0.04–1.00)
Last use >=6 years previously	2/710	1/395	0.09 (0.02–0.39)
Progesterone-alone			
Current users	3/9	0/0	—
Last use 1–3 years previously	0/23	0/0	—
Last use 4-5 years previously	0/19	0/0	—
Last use >=6 years previously	0/32	0/0	—
Estrogen and progesterone combination			
Current users	0/0	0/0	—
Last use 1–3 years previously	0/0	0/0	—
Last use 4-5 years previously	0/0	0/0	—
Last use >=6 years previously	0/0	0/0	—
Mixed type^†^			
Current users	5/69	7/246	1.00
Last use 1–3 years previously	1/69	8/192	0.91 (0.38–2.15)
Last use 4-5 years previously	0/118	0/614	—
Last use >=6 years previously	16/836	11/1,081	0.37 (0.19–0.73)

*HT refers to hormonal therapy.

^†^Mixed type refers to the estrogen-alone (E-alone); estrogen together with progesterone (E + P); other preparations, which included progesterone only and vaginal and other local treatments and combinations of the above preparation types.

aHR refers to the hazard ratios adjusted by age and number of chronic diseases.
